# Neuromuscular performance of the glenohumeral joint in young female tennis players: a cross-sectional study

**DOI:** 10.3389/fphys.2026.1854908

**Published:** 2026-06-17

**Authors:** Tomasz Waldziński, Aleksandra Durzyńska, Bartłomiej Niespodziński, Jan Mieszkowski, Andrzej Kochanowicz

**Affiliations:** 1 Faculty of Health Sciences, University of Lomza, Łomża, Poland; 2 Department of Biological Foundations of Physical Education, Faculty of Health Sciences and Physical Education, Kazimierz Wielki University, Bydgoszcz, Poland; 3 Department of Gymnastics and Dance, Gdańsk University of Physical Education and Sport, Gdańsk, Poland

**Keywords:** athletes, coactivation, EMG, explosive strength, RTD, shoulder

## Abstract

**Introduction:**

The glenohumeral joint plays a key role in force generation during tennis strokes. Neuromuscular adaptations in this joint are critical for performance and injury prevention, particularly during rapid phases of movement. However, there is limited research on these adaptations in young female tennis players. Therefore, this study aimed to examine differences in glenohumeral neuromuscular performance between female tennis players and untrained girls aged 11–14 years.

**Methods:**

A cross-sectional study included 67 participants: 33 female tennis players (aged 11–12 and 13-14) and 34 age-matched controls. Isometric peak torque (PKTQ), rate of torque development (RTD), and surface electromyography (SEMG) were assessed for internal (IR) and external (ER) rotation. A two-way ANOVA and MANOVA were used for statistical analysis.

**Results:**

Tennis players showed significantly higher normalized PKTQ during IR (18.1%, p < 0.01) and ER (10.1%, p < 0.05), and greater RTD during ER (absolute: 28.4%, p < 0.01; normalized: 32.2%, p < 0.01) compared to controls. A group-by-age interaction showed greater normalized peak RTD during ER in 11–12-year-old tennis players compared with their untrained counterparts. SEMG revealed greater posterior deltoid activation during ER at 50% MVIC (+15.7%, p < 0.05) and lower pectoralis major coactivation during ER at 50% MVIC (−40.4%, p < 0.01) in tennis players. Additionally, tennis players exhibited a higher IR/ER ratio (13.3%, p < 0.05).

**Conclusions:**

Long-term tennis training may be associated with specific neuromuscular adaptations in the glenohumeral joint, particularly higher RTD during ER in younger athletes. These findings may be relevant to stroke velocity and shoulder stability and highlight the importance of age-specific training for the development of optimal glenohumeral strength and injury prevention.

## Introduction

Tennis strokes rely on force transfer through the kinetic chain, progressing from the lower limbs and trunk to the shoulder complex and upper limb, ultimately generating racket head velocity at ball impact. As the proximal initiator of upper limb motion, the glenohumeral joint plays a critical role in biomechanics of the entire upper limb including power generation and precision (Cirstea et al., 2003). In this context, glenohumeral joint movements are among the most significant contributors to the total force production during a tennis stroke (Blache et al., 2017). It has been observed that the glenohumeral joint accounts for approximately 60% of the upper limb’s contribution to racket head speed during the preparatory phase of a forehand stroke (Takahashi et al., 1996). The importance of the glenohumeral joint appears to be critical in relation to stroke technique (Takahashi et al., 1996), stroke velocity (Seeley et al., 2011), and level of athletic proficiency (Landlinger et al., 2010). Repetitive motor activities associated with specific stroke techniques induce a range of muscle activity changes (Chow et al., 2007; De Smedt et al., 2007; Eygendaal et al., 2007; Alizadehkhaiyat and Frostick, 2015), which may contribute to the development of specific neuromuscular adaptations depending on age and skill level in tennis (Zemková and Kováčiková, 2022).

Elite tennis players often begin intensive training in early childhood, which coincides with a critical period of rapid neuromuscular development (Cools et al., 2014). Long-term exposure to repetitive tennis-specific loading may contribute to adaptations in shoulder neuromuscular performance during internal and external rotation movements (Kibler et al., 2018). Among young tennis players, an increase in external rotation (ER) strength of the dominant glenohumeral joint is commonly observed (Saccol et al., 2010; Cigercioglu et al., 2021), whereas in adult athletes, greater muscular strength is often found in internal rotation (IR) of the dominant limb compared to the non-dominant one (Ng and Kramer, 1991; Ellenbecker and Roetert, 2003; Gozlan et al., 2006; Silva et al., 2006; Brito et al., 2022). Furthermore, higher muscle strength values in both IR and ER are characteristic of players competing at the national level compared to those participating in amateur tournaments (Johansson et al., 2022). On the other hand, the development of glenohumeral rotator strength across different age groups of tennis players is often accompanied by a reduction in IR range of motion and a decreased total range of motion in the dominant arm compared to the non-dominant one. This may increase the risk of injuries among athletes (Vad et al., 2003; Schmidt-Wiethoff et al., 2004; Borich et al., 2006; Marcondes et al., 2013).

Existing evidence indicates that strength development in young female tennis players may be influenced more by increases in body size and lean mass than by pronounced neuromuscular adaptations typically observed in males (Fernandez-Fernandez et al., 2019; Tooth et al., 2022). This pattern may reflect a complex and still insufficiently explored interaction between morphological growth and neuromuscular coordination. It is also important to note that many studies on muscle strength in young tennis players lack control groups, which limits the ability to distinguish training-related adaptations from typical development in untrained peers (Fernandez-Fernandez et al., 2019; Johansson et al., 2022; Tooth et al., 2022). Notably, previous research in young female tennis players demonstrated that the most pronounced tennis-related differences in upper-limb proprioception were observed in the glenohumeral joint, where tennis players outperformed their untrained peers in joint position sense and selected force-sense tasks (Niespodziński et al., 2024). These observations provide further justification for examining glenohumeral neuromuscular performance more directly, using measures of force, rate of torque development (RTD), and surface electromyography (SEMG). Despite these insights, studies addressing the influence of training on glenohumeral neuromuscular performance and coordination in youth tennis remain limited, particularly in young female players.

Accordingly, the aim of this study was to compare neuromuscular performance and muscle activity during IR and ER between female tennis players aged 11–12 and 13–14 years and their age-matched untrained peers.

## Materials and methods

### Study overview

This study employed a cross-sectional design. A purposive sampling method was used to recruit participants, resulting in two groups of girls aged 11–14 years: one consisting of individuals with several years of systematic tennis training, and a comparison group of untrained peers. To address the study objectives, neuromuscular performance and SEMG assessments were conducted for the glenohumeral joint.

### Participants

A total of 72 girls were enrolled in the study, including 36 tennis players and 36 controls. Five girls (3 tennis players and 2 controls) were excluded, resulting in a final sample of 67 participants. An *a priori* sample size calculation was performed using G*Power software ver. 3.1.9.7. (Franz Faul et al., Universität Kiel, Kiel, Germany). Assuming a large effect size for the planned group- and age-related effects (f = 0.40; η² = 0.14), α = 0.05, and statistical power of 0.80, the minimum required total sample size was 52 participants. Accordingly, the final analytical sample exceeded this requirement. The study group comprised 33 female tennis players who actively competed at the regional and national levels, in accordance with the classification system of the Polish Tennis Association. Within this group, the younger subgroup consisted of 17 players aged 11–12 years, classified in the U12 category, whereas the older subgroup consisted of 16 players aged 13–14 years, classified in the U14 category. Players in both subgroups were ranked within the top 40 of the Polish Tennis Association ranking in the year of the study. Inclusion criteria for the tennis group were regular tennis training for at least two years and participation in at least 12 national or international tennis tournaments before enrollment in the study. The control group comprised 34 non-athlete girls matched for age, including 18 girls aged 11–12 years and 16 girls aged 13–14 years. The characteristics of the study groups and subgroup allocation are presented in **Table 1**. Body height was measured without shoes to the nearest 0.1 cm using a calibrated stadiometer (Model 213, SECA, Hamburg, Germany). Body mass was measured using a body composition analyzer (InBody 720, Biospace Co., Ltd., Seoul, Korea).

**Table 1:** Selected characteristics of untrained girls and female tennis players in each age category (mean ± standard deviation).

Variable	Control		Tennis players	
	11–12 years old (n = 18)	13–14 years old (n = 16)	11–12 years old (n = 17)	13–14 years old (n = 16)
Age (years)	11.68 ± 0.70	13.94 ± 0.62	11.35 ± 0.82	14.14 ± 0.73
Body height (cm)	152.83 ± 8.95	162.87 ± 4.85	153.62 ± 8.74	163.58 ± 6.45
Body mass (kg)	44.40 ± 10.58	55.75 ± 10.41	43.28 ± 7.25	51.57 ± 7.45
BMI (kg·m^-2^)	18.82 ± 3.12	20.99 ± 3.70	18.24 ± 2.08	19.20 ± 1.82
Training experience (years)	–	–	5 ± 0.5	7 ± 0.75
Weekly training volume (h/week)	–	–	7-11	9-13

BMI, body mass index. Weekly training volume refers to the typical weekly range of training hours at the female tennis players’ current stage of tennis training.

The tennis players were recruited from leading tennis clubs in the Pomeranian, Podlaskie, and Warmian-Masurian provinces of Poland. Recruitment was conducted in consultation with coaches from seven regional clubs to identify players who met the predefined sport-specific inclusion criteria. On average, players aged 11–12 years were classified at level 6 ± 1.97, whereas those aged 13–14 years were classified at level 4 ± 1.12 on the International Tennis Number scale (ITN), on which level 1 represents an elite/professional player and level 10 represents a beginner player (Johnston et al., 2004). The ITN was provided by the players’ coaches based on regular club assessments conducted during the study period. The control group was recruited from local primary schools and included girls who did not participate in any structured sports training outside regular school-based physical education classes. Both the tennis players and the control participants attended regular school-based physical education classes consisting of four 45-minute sessions per week.

The study was conducted during the preparatory period of the tennis players’ annual training cycle. For the tennis group, an interruption in training lasting at least one month within the two months preceding the study was adopted as an exclusion criterion. For both groups, additional exclusion criteria included any diagnosed neurological, neuromuscular, or musculoskeletal condition that could affect upper-limb motor function or the safe performance of maximal shoulder exertion, such as peripheral nerve disorders, myopathies, current shoulder pain or injury, previous shoulder injury or surgery, or acute injury of the tested upper limb. Eligibility screening was conducted before testing using a structured questionnaire on health status, previous injury history, including shoulder injuries, and training history, completed with the participants and their legal guardians. Three excluded tennis players (one 11–12-years old and two 13–14-years old) did not attend the testing session due to temporary health-related reasons, including viral infection or muscle soreness. The remaining two individuals (13–14-years old control group) were excluded due to exceeding the outlier criteria.

The study was conducted in accordance with the principles of the Declaration of Helsinki and was approved by the Bioethics Committee of the Regional Medical Chamber in Gdańsk (protocol code: KB-25/20; approval date: 27 October 2020). Written informed consent was obtained from all participants and their legal guardians prior to participation.

### Neuromuscular performance

Each participant completed a series of tests to assess muscle strength using a Biodex System 4 Pro isokinetic dynamometer (Biodex Shirley Corporation, Shirley, NY, USA). The protocol included the measurement of peak torque (PKTQ) and its RTD under isometric conditions, using a sampling frequency of 100 Hz. This allowed for an evaluation of force production potential and its differences related to the tennis training and age, specifically in terms of glenohumeral maximal static strength capacity (PKTQ) and explosive neuromuscular performance (RTD), which are functionally relevant to upper-limb control and force transfer during tennis-specific stroke actions (Baiget et al., 2016, Baiget et al., 2022). The reliability of this device has been repeatedly validated across various studies, demonstrating good to excellent test-retest reproducibility depending on the functional joint complex being assessed (Holmbäck et al., 1999; Meeteren et al., 2002; Drouin et al., 2004; Zawadzki et al., 2010).

Maximal voluntary isometric contraction (MVIC) tests were performed in the glenohumeral joint and assessed the muscle strength of IR and ER muscles. Measurements were conducted according to the manufacturer’s guidelines. The participants were seated in an adjustable seat of the device with the backrest adjusted to their body dimensions. The glenohumeral joint was positioned at 90° of abduction in the frontal plane, with the elbow flexed at 90° and stabilized to the dynamometer arm. The hand and forearm were in a neutral position, holding the dynamometer handle, and the device’s rotational axis was aligned with the participant’s olecranon process. The glenohumeral joint was positioned at 0° of axial rotation, defined as the neutral position between external and internal rotation.

Prior to testing, participants were familiarized with the procedure and performed practice contractions for each rotation direction before the recorded trials. Testing began only after participants understood the task and were able to perform the contraction without visible compensatory movements. Measurements were performed on the dominant upper limb, determined by hand preference for writing. Participants were instructed to perform three 3-s MVICs for both IR and ER of the glenohumeral joint. During each trial, participants were encouraged to reach PKTQ as quickly as possible and maintain it until cued to stop by the examiner. Verbal encouragement was consistently provided to elicit maximal effort (McNair et al., 1996; Sahaly et al., 2003). A one-minute rest interval between trials was implemented to allow for muscle recovery (Celes et al., 2009).

Adjacent joints were stabilized using leather straps to prevent compensatory movements. For further analysis, the trial producing the highest torque for each joint action was selected. The following muscle strength characteristics were analyzed: PKTQ, peak RTD, RTD normalized to PKTQ, and RTD values calculated over successive time intervals: 0–10, 0–20, 0–30, 0–40, 0–50, 0–60, 0–70, 0–80, 0–90, 0–100, 0–110, 0–120, 0–130, 0–140, 0–150, 0–160, 0–170, 0–180, 0–190, and 0–200 ms. PKTQ values were also normalized to body mass and expressed as a ratio of internal to external rotation (IR/ER) performance. The IR/ER ratio was assessed to evaluate whether neuromuscular adaptations favor the dominance of one muscle group over another, which may enhance sport-specific performance while simultaneously increasing injury risk, particularly in highly mobile joints such as the glenohumeral joint (Moreno-Pérez et al., 2018). In order to avoid inter-rater bias, neuromuscular performance evaluation was performed by the same trained investigator.

### Surface electromyography

A bipolar differential SEMG technique was used to analyze the neuromuscular characteristics of glenohumeral performance. Electrode (Sorimex, Toruń, Poland; Ag/AgCl 1-cm² active electrode surface) placement followed the guidelines of the Surface Electromyography for the Non-Invasive Assessment of Muscles (SENIAM) project (Hermens et al., 1999) project and Cram’s Introduction to Surface Electromyography (Criswell, 2011). The skin area for electrode placement was prepared by removing hair if necessary, degreasing with an alcohol solution, and gently abrading (Spes Medica S.p.A., Genova, Italy) the stratum corneum to reduce impedance.

Electromyographic signals were recorded using the TeleMyo DTS system (Noraxon, Scottsdale, AZ, USA), featuring a gain of up to 500×, 16-bit analog-to-digital conversion, input impedance greater than 100 MΩ, a common mode rejection ratio exceeding 100 dB, a sampling rate of 1500 Hz, and a band-pass filter set between 10–500 Hz. The acquired signals were processed, stored, and analyzed using MyoResearch 1.08 software (Noraxon, Scottsdale, AZ, USA).

Muscle activity was assessed during MVIC and submaximal contractions at 20% and 50% of MVIC for both IR and ER. The selected submaximal contraction intensities reflected different neuromuscular activation demands including differences in motor unit recruitment and discharge rates (de Luca et al., 1996; Hassan et al., 2019; Kowalski and Anita, 2020). Lower-force contractions (20% MVIC) are considered particularly sensitive to fine motor control, force steadiness, and low-threshold motor unit activity, whereas moderate-force contractions (50% MVIC) involve greater neural drive and recruitment of higher-threshold motor units. Previous studies (de Luca et al., 1996) have shown, among others, that the rate of decrease in mean motor unit firing rate is related to force level (30% vs. 50% MVIC), and that contraction intensity-specific differences in neuromuscular activation patterns may occur between 20% and 50% MVIC in the presence of mental fatigue (Kowalski and Anita, 2020). Moreover, different muscles may exhibit distinct activation patterns across various contraction intensities (10–50% MVIC), although this may also reflect differences in the motor units detectable using current methodological approaches (Hassan et al., 2019). Submaximal SEMG assessment gives insight into training- and maturation-related neuromuscular adaptations (Grosset et al., 2008; Vila-Chã et al., 2010). Submaximal tests were performed five minutes after MVIC testing. Visual feedback was provided on a monitor, showing both the real-time torque output and the target torque level (20% or 50% of MVIC). Participants were instructed to reach and maintain the specified torque for 3 seconds. The order of target levels (20% or 50% MVIC) was randomized. Each test was performed three times, with a one-minute rest interval between trials to ensure recovery.

Given the inherent variability of the SEMG signal, the mean value from three consecutive trials was used for subsequent analysis. The raw SEMG signal obtained during testing was smoothed using the root mean square (RMS) algorithm with a 100-ms time window and was full-wave rectified.

For further analysis, the average RMS amplitude was extracted from the central 2-second period of the MVIC, following the recommendations of Criswell (2011). This value was also used to normalize the RMS amplitude (NRMS) obtained during submaximal contractions at 20% and 50% MVIC.

In addition to analyzing SEMG activity of the agonist muscles during contraction, a coactivation index (CI) was calculated to assess the level of antagonist muscle activation according to the formula (Ervilha et al., 2012):


$$\text{CI}=\frac{Surface\;electromyographic\;activity\;of\;the\;muscle\;acting\;as\;an\;antagonist}{Surface\;electromyographic\;activity\;of\;the\;muscle\;acting\;as\;an\;agonist}$$

Coactivation refers to the simultaneous activation of an antagonist muscle during an agonist muscle action. It is suggested to provide precision and joint stability, though its high level may be detrimental to force production or movement efficiency (Woods et al., 2023).

The muscles analyzed in this study included: the anterior deltoid, latissimus dorsi, and the sternocostal portion of the pectoralis major, which served as the agonists for IR; as well as the posterior deltoid and infraspinatus, which acted as the agonists for ER. To eliminate inter-rater variability, a single trained investigator performed all SEMG preparations and measurements.

### Statistical analysis

Descriptive statistics, including means and standard deviations, were calculated for all variables. A two-way analysis of variance (ANOVA) was performed with group (tennis players vs. untrained) and age (11–12 vs. 13–14 years) as factors. This approach enabled assessment of the differentiating effect of tennis training in relation to biological development across two stages of the athlete training pathway (i.e., the U12 and U14 categories).

For the analysis of RTD, due to the high number of closely related time-point variables, intervals were grouped into two categories: early RTD (0–10, 0–20, 0–30, 0–40, 0–50, 0–60, 0–70, 0–80, 0–90, and 0–100 ms) and late RTD (0–110 to 0–200 ms, in 10 ms increments). The grouping of variables was performed based on evidence indicating that early (<100 ms) and late (>100 ms) RTD reflect partially different physiological determinants. Specifically, early RTD is considered to depend predominantly on neural factors, including motor unit recruitment speed and discharge rate, whereas later RTD is more strongly influenced by maximal force-generating capacity and musculotendinous properties (Andersen and Aagaard, 2006; Maffiuletti et al., 2016; Del Vecchio et al., 2019). These grouped variables were subjected to multivariate analysis of variance (MANOVA), using the same two factors (group and age). Multivariate effects were evaluated using Wilks’ lambda (Wilks’ λ). Only if significant main or interaction effects were identified in the MANOVA were further univariate ANOVAs conducted on individual time intervals.

Assumptions of normality and homogeneity of variance were verified using the Shapiro-Wilk and Levene’s tests, respectively. In the event of significant main effects or interactions, *post hoc* Tukey’s tests for unequal sample sizes were applied to identify specific between-group differences. Tukey’s *post hoc* test was used to account for multiple pairwise comparisons within each ANOVA model. For RTD time-interval analyses, MANOVA was used as a multivariate gatekeeping procedure before conducting univariate ANOVAs.

Effect sizes for main effects and interactions were calculated using eta-squared (η²), with the thresholds for interpretation set at: small (η² = 0.01), medium (η² = 0.06), and large (η² > 0.14) (Cohen, 1988; Levine and Hullett, 2002). Outliers exceeding three standard deviations from the group mean were excluded from the analysis. The level of statistical significance was set at α = 0.05. All statistical procedures were conducted using Statistica 13.3 software (StatSoft Inc., Tulsa, OK, USA).

## Results

Peak torque and torque kinetic characteristics during glenohumeral IR and ER are presented in **Table 2**. Regardless of age category, female tennis players demonstrated significantly higher values of both absolute PKTQ (+14.8%, F_1,63_ = 5.42, *p* < 0.05, η² = 0.08) and PKTQ normalized to body mass (+18.1%, F_1,63_ = 13.06, *p* < 0.01, η² = 0.17) during IR of the glenohumeral joint compared to untrained peers. In the case of ER, all tennis players exhibited significantly higher normalized PKTQ values (+10.1%, F_1,63_ = 4.42, *p* < 0.05, η² = 0.07), alongside significantly greater absolute (+28.4%, F_1,63_ = 9.44, *p* < 0.01, η² = 0.13) and normalized peak RTD (32.2%, F_1,63_ = 13.41, *p* < 0.01, η² = 0.17) than untrained girls.

**Table 2:** Peak torque and torque kinetic characteristics during internal and external rotation of the glenohumeral joint.

Variable	Glenohumeral rotation	Control		Tennis players	
		11–12 years old (n = 18)	13–14 years old (n = 16)	11–12 years old (n = 17)	13–14 years old (n = 16)
PKTQ (Nm)	Internal	14.75 ± 4.02	21.28 ± 4.73	17.98 ± 4.12	23.67 ± 6.44
	External	16.24 ± 3.89	20.1 ± 4.54	17.30 ± 3.28	20.95 ± 4.89
PKTQ/BM (Nm·kg^-1^)	Internal	0.34 ± 0.08	0.38 ± 0.07	0.42 ± 0.10	0.45 ± 0.08
	External	0.37 ± 0.06	0.36 ± 0.07	0.40 ± 0.08	0.40 ± 0.08
PKTQ ratio	Internal/External	0.91 ± 0.15	1.06 ± 0.14	1.05 ± 0.21	1.17 ± 0.38
Peak RTD (Nm·s^-1^)	Internal	98.69 ± 45.26	178.50 ± 123.73	142.24 ± 48.28	186.18 ± 90.18
	External	190.91 ± 113.78	371.32 ± 108.97	323.89 ± 115.38	429.82 ± 158.45
Peak RTD/PKTQ, (Nm·s^-1^·Nm^-1^)	Internal	6.87 ± 2.85	8.39 ± 5.47	8.03 ± 2.63	7.75 ± 2.87
	External	11.42 ± 5.23	18.51 ± 4.20	18.30 ± 4.51*	20.28 ± 5.01

PKTQ, peak torque; BM, body mass; RTD, rate of torque development. *Significant difference vs 11–12 years old controls at p < 0.05.

Regardless of tennis training status, girls aged 13–14 years demonstrated significantly higher absolute PKTQ values during both IR (+27.5%, F_1,63_ = 24.50, *p* < 0.01, η² = 0.28) and ER (+18.5%, F_1,63_ = 13.62, *p* < 0.01, η² = 0.18) compared to girls aged 11–12 years. Furthermore, significantly greater absolute RTD values were observed in the older group during IR (+34.6%, F_1,63_ = 9.31, *p* < 0.01, η² = 0.13), as well as significantly higher absolute (+37.5%, F_1,63_ = 21.13, *p* < 0.01, η² = 0.25) and normalized RTD values (+32.7%, F_1,63_ = 14.75, *p* < 0.01, η² = 0.19) during ER relative to their younger counterparts.

Additionally, the ratio of IR/ER was significantly higher (+13.3%, F_1,63_ = 4.74, *p* < 0.05, η² = 0.07) in tennis players (1.11 ± 0.31) compared to the control group (0.98 ± 0.19), and the older group (1.12 ± 0.29) had significantly higher (15.5%, F_1,63_ = 5.48, *p* < 0.05, η**^2^** = 0.08) IR/ER ratio compared to the younger group (0.97 ± 0.19). These results reflect the higher muscle strength of the internal rotators over the external rotators in both the tennis players and the older group.

Finally, a significant interaction effect between group and age was found, with female tennis players aged 11–12 years demonstrating a significantly greater normalized peak RTD during ER of the glenohumeral joint (+62.7%, F_1,63_ = 4.68, *p* < 0.05, η² = 0.07) compared to their untrained counterparts (**Table 2**).

**Figure 1:**
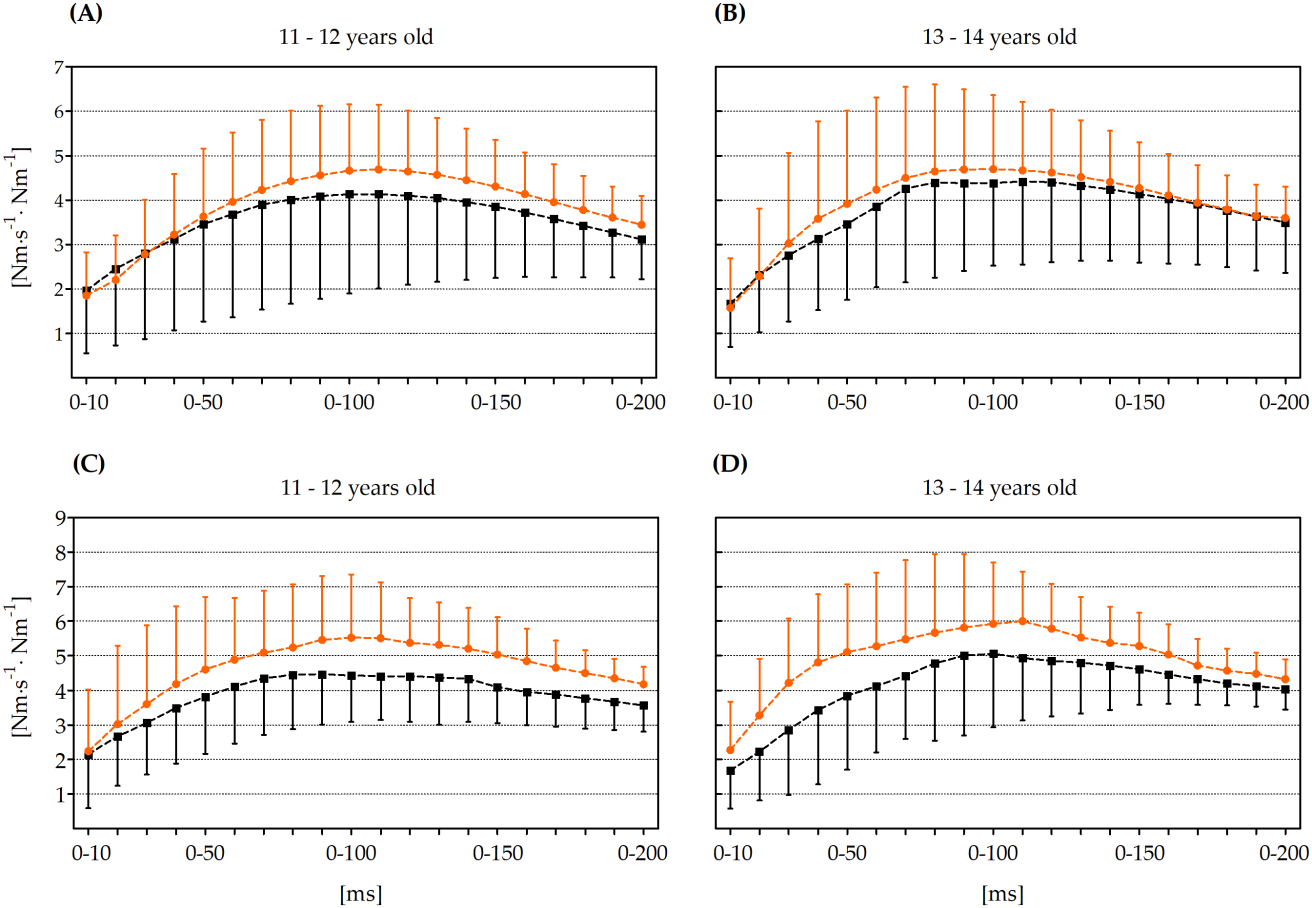
Mean ± standard deviation of RTD normalized to peak torque during isometric glenohumeral internal and external rotation in female tennis players (orange) and untrained controls (black). **(A)** Internal rotation in girls aged 11–12 years; **(B)** internal rotation in girls aged 13–14 years; **(C)** external rotation in girls aged 11–12 years; and **(D)** external rotation in girls aged 13–14 years. The x-axis presents consecutive time intervals from torque onset (0–10 to 0–200 ms).

The means and standard deviations of normalized RTD values across individual time intervals during glenohumeral IR and ER are presented in **Figure 1**. Although a significant main effect of group was observed for early-phase IR RTD (Wilks’ λ = 0.70, F_10,54_ = 2.45, *p* < 0.05, η² = 0.30), *post hoc* analysis did not reveal statistically significant differences at any specific time interval.

**Figure 2:**
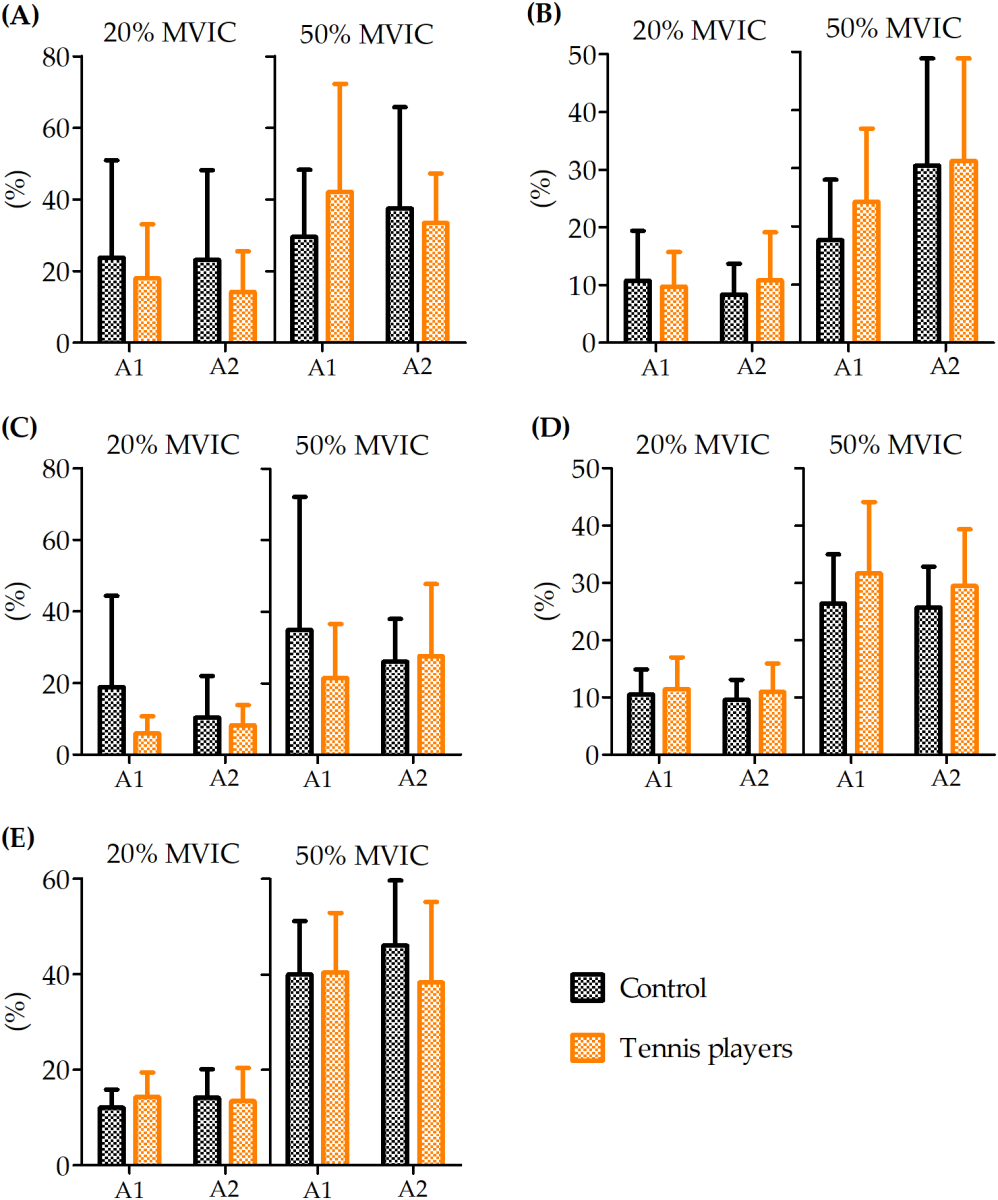
Mean ± standard deviation of normalized muscle activity of selected agonist muscles during glenohumeral internal rotation **(A)**, anterior deltoid; **(B)**, latissimus dorsi; **(C)**, pectoralis major) and external rotation **(D)**, posterior deltoid; **(E)**, infraspinatus) at 20% and 50% of maximal voluntary isometric contraction. A1, group of 11–12-year-olds; A2, group of 13–14-year-olds.

Mean values and standard deviations of SEMG signals of the evaluated agonist muscles during 20% and 50% of MVIC in glenohumeral IR and ER are presented in **Figure 2**. ANOVA revealed that, irrespective of age, female tennis players exhibited significantly higher normalized activation of the posterior deltoid muscle during the 50% MVIC task in ER compared to untrained girls (+15.7%, F_1,63_ = 4.02, *p* < 0.05, η² = 0.06). Conversely, during IR, significantly lower SEMG activity of the latissimus dorsi muscle at 50% MVIC was observed in girls aged 11–12 years compared to those aged 13–14 years (−33.1%, F_1,63_ = 7.00, *p* < 0.01, η² = 0.10), regardless of training status.

**Figure 3:**
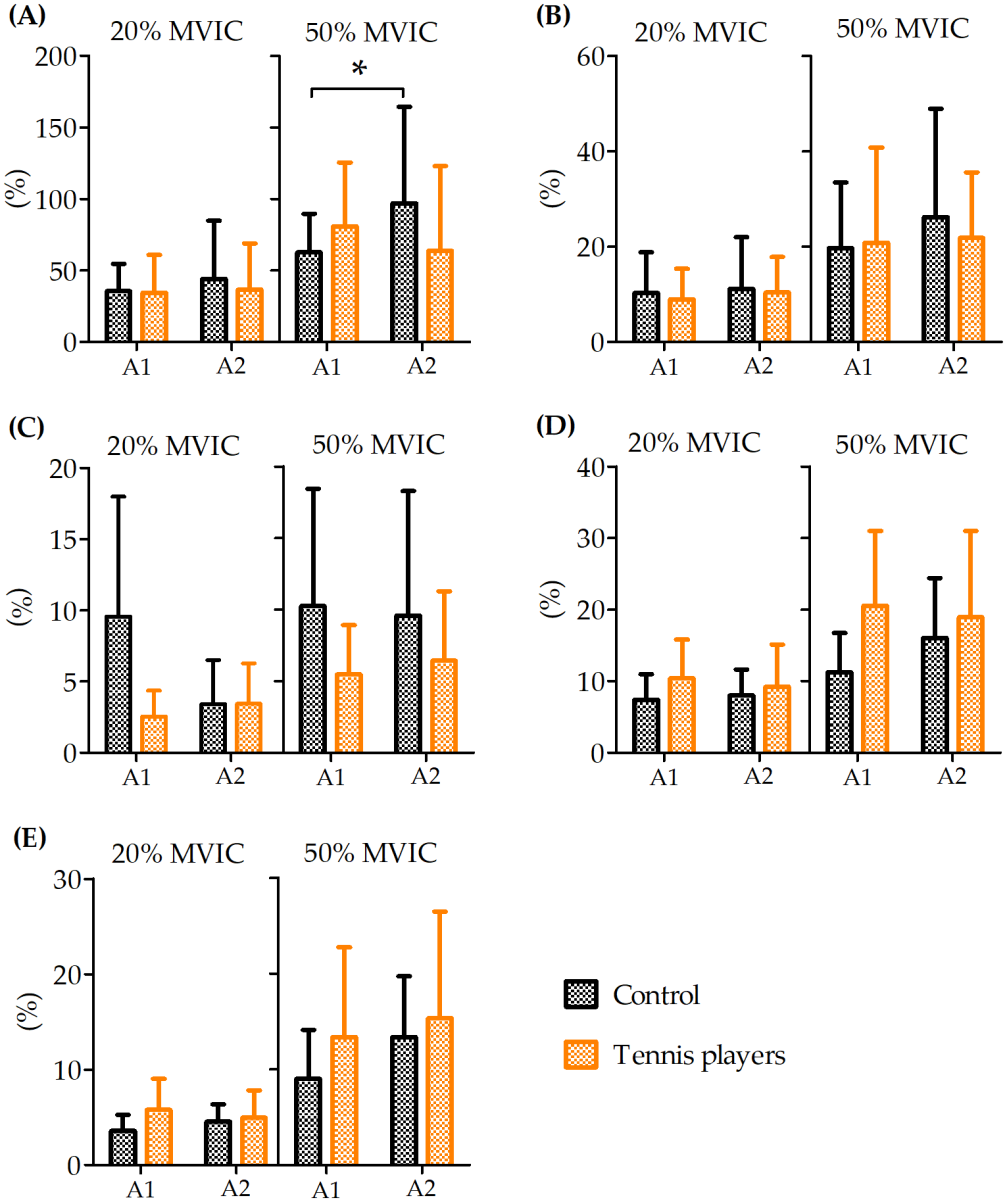
Mean ± standard deviation of the coactivation index of selected antagonist muscles during glenohumeral external rotation **(A)**, anterior deltoid; **(B)**, latissimus dorsi; **(C)**, pectoralis major) and internal rotation **(D)**, posterior deltoid; **(E)**, infraspinatus) at 20% and 50% of maximal voluntary isometric contraction. A1, group of 11–12-year-olds; A2, group of 13–14-year-olds. *Significant difference at p < 0.01.

The mean values and standard deviations of the CI for the evaluated antagonist muscles during 20% and 50% of MVIC in glenohumeral IR and ER are presented in **Figure 3**. ANOVA revealed that, irrespective of age, female tennis players exhibited significantly higher CI values during IR at 50% MVIC for the posterior deltoid (+31.6%, F_1,63_ = 7.13, *p* < 0.01, η² = 0.10) and for the infraspinatus during both the 20% (+24.8%, F_1,63_ = 4.62, *p* < 0.05, η² = 0.07) and 50% MVIC tasks (+26.6%, F_1,63_ = 4.16, *p* < 0.05, η² = 0.06), compared to untrained girls. In contrast, the CI of the pectoralis major during ER at 50% MVIC was significantly lower (−40.4%, F_1,63_ = 5.91, *p* < 0.05, η² = 0.09) in tennis players than in untrained girls.

Additionally, a significant group-by-age interaction effect was observed for the anterior deltoid during ER at 50% MVIC. *Post hoc* analysis indicated significantly higher CI values in untrained girls aged 13–14 years compared to their 11–12-year-old untrained peers (+35.4%, F_1,63_ = 4.15, *p* < 0.05, η² = 0.06).

## Discussion

The study aimed to analyze differences in neuromuscular performance during IR and ER of the glenohumeral joint between female tennis players aged 11–12 and 13–14 years and their untrained peers. The analysis focused on muscle strength-related parameters, including PKTQ and RTD, as well as activity of agonist and antagonist muscles.

One of the main findings of the present study was that tennis players from both age groups demonstrated significantly higher peak RTD values only during ER compared to their untrained peers. Moreover, tennis players aged 11–12 years exhibited substantially higher peak RTD values normalized to PKTQ during glenohumeral ER than untrained controls. These results were also comparable to those observed in the older tennis players (13–14 years). This finding suggests that long-term tennis training may be associated with more advanced development of ER muscle function, particularly in terms of RTD.

These differences could be partially influenced by pubertal changes; however, no significant differences in body height or body mass were found between 11–12-year-old tennis players and their untrained counterparts. Nevertheless, given the cross-sectional design and the recruitment of competitive players ranked within the top 40 of the Polish Tennis Association, the potential influence of selection bias cannot be excluded. Thus, the higher ER RTD observed in younger tennis players may partly reflect pre-existing neuromuscular predispositions in addition to tennis training experience.

Currently, there is a scarcity of studies investigating RTD during IR and ER of the glenohumeral joint in young tennis players. One of the few existing studies (Baiget et al., 2022) similarly indicated that the highest rate of force development occurred within the 0–150 ms window (including subintervals: 0–30, 0–50, 0–90, 0–100, 0–150, 0–200, and 0–250 ms). In contrast, the present study narrowed this time window more precisely to 0–100 and 0–110 ms.

In the present study, both tennis players and untrained controls exhibited higher RTD values during ER than during IR. This differs from the findings of Baiget et al. (2022), who reported higher peak RTD values during IR. Although this discrepancy may partly reflect differences in the study populations, with younger girls, whereas Baiget et al. (2022) examined older adolescents. However, differences in the testing setup appear to be a more plausible explanation. In both studies, the glenohumeral joint was assessed with the shoulder abducted to 90° and the elbow flexed to 90°. However, Baiget et al. (2022) evaluated torque production from a position of 90° ER, whereas in the present study testing was performed from a neutral rotation position. Because 90° ER approximates the end range of motion, this configuration may mechanically limit rapid torque generation. A similar effect was reported by Hager et al. (2020), who showed that peak RTD was substantially lower when contractions were performed near the end range than from a neutral joint position.

Previous research suggests that the ability to generate force rapidly, particularly within the glenohumeral joint, may contribute to stroke velocity in tennis, both in youth and elite adult players (Baiget et al., 2022). In tennis strokes such as the serve and groundstrokes (forehand and backhand), IR and ER contribute to different phases of movement rather than being exclusively associated with a single stroke type. ER is particularly important during the preparatory phase, when the shoulder is positioned to allow subsequent acceleration of the upper limb and racquet, whereas IR contributes substantially to the forward acceleration phase of the stroke (Ryu et al., 1988; Rogowski et al., 2011; Rota et al., 2012; Saeki et al., 2025). Since forehand strokes are used more frequently than backhand strokes during competitive play (Fernandez-Fernandez et al., 2010; Reid et al., 2016; Filipcic et al., 2017), repeated exposure to these phase-specific rotational demands may help explain why training-related differences in the present study were particularly evident for ER RTD.

One of the key factors influencing the RTD is the PKTQ that an individual is able to generate. In the case of ER, RTD normalized to PKTQ remained significantly higher in tennis players compared to untrained peers (**Table 2**). This suggests that additional mechanisms, beyond maximal muscle strength, may contribute to RTD performance, such as muscle-tendon mechanical properties, the force-velocity relationship, and neuromuscular control in the form of motor unit recruitment speed and discharge rate (Del Vecchio et al., 2019; Hager et al., 2020).

In contrast, although peak RTD did not differ significantly between tennis players and untrained girls, the group effect observed for early-phase IR RTD (**Table 2**) may be partly related to higher absolute and body mass-normalized IR PKTQ in tennis players, which was also reflected in their higher IR/ER ratio.

Only a limited number of studies have compared glenohumeral strength parameters between tennis players and untrained individuals. In line with the present findings, previous research generally indicates higher glenohumeral muscle strength in tennis players than in untrained peers (Myers et al., 2024), with strength adaptations reported in children and adolescents (Tooth et al., 2022), adults (Ellenbecker, 1991; Elliott, 2006), as well as older adults (Yoshiko et al., 2022). The greater maximal IR strength (e.g. measured in form of PKTQ) observed in tennis players is commonly attributed to the repetitive demands of the serve and forehand performed over several years of training (Elliott, 2006; Brito et al., 2022). By comparison, the more moderate increase in maximal ER strength may reflect the functional need to maintain dynamic shoulder stability as training loads progressively increase during athletic development (Saccol et al., 2010). The presented results, in line with previous studies, suggest that long-term tennis training induces changes in PKTQ of both IR and ER typically before the age of 11.

Tennis training-related adaptations in glenohumeral strength are also commonly discussed in relation to the IR/ER ratio. Recommended IR/ER ratios have been reported to range from 1.33 to 1.52, corresponding to at least a 66% ER/IR ratio, to ensure that the IR strength does not exceed 1.5 times that of the ER strength (Ellenbecker and Davies, 2000). Exceeding this threshold may increase the risk of shoulder injury, particularly in overhead athletes (Moreno-Pérez et al., 2018). In the present study, the IR/ER ratio in tennis players was higher than in untrained peers, but remained below the 1.5 threshold and was consistent with values previously reported in 11–14-year-old tennis players (Ellenbecker, 1992) and in female tennis players in the U13 and U15 age categories (Fernandez-Fernandez et al., 2019). However, Johansson et al. (2022), reported IR/ER values reaching 1.49 in adolescent female players, with a considerable number of players older than 14 years exceeding the recommended limit. Such variability is likely related to methodological differences, including the use of handheld versus isokinetic dynamometry, differences in glenohumeral joint positioning during testing, and variation in participant age and training experience.

The comparison of girls aged 11–12 and 13–14 years provided insight into glenohumeral neuromuscular characteristics across two stages of youth tennis participation. Previous longitudinal observations suggest that motor coordination and tennis skills development in young players are age- and task-dependent, with sport-specific progress becoming increasingly evident during this period (Waldziński et al., 2024). Consistent with previous studies, absolute PKTQ (Sunnegårdh et al., 1988; Pitcher et al., 2012; Johansson et al., 2022) and RTD (Asai and Aoki, 1996; Grosset et al., 2005; Falk et al., 2009) increase with age in the present sample. However, older girls did not differ from younger girls in body mass-normalized PKTQ, regardless of training status, which is consistent with observations in young tennis players across comparable developmental stages, including male tennis players aged 7–13 years (Gillet et al., 2017), female players below and above 14 years of age (Johansson et al., 2022), players in the U13 and U15 categories (Fernandez-Fernandez et al., 2019), and players aged 12–17 and 18–21 years (Ellenbecker and Roetert, 2003). Overall, these findings suggest that, within the studied age range, the most distinct training-related difference in muscle strength characteristics was evident for ER RTD rather than maximal torque alone.

Because both RTD and PKTQ are influenced by neural factors, including motor unit recruitment and discharge rates (Maffiuletti et al., 2016), SEMG analysis provides an important complementary perspective on glenohumeral neuromuscular performance. Muscle activation patterns in relation to force or torque generation provide valuable insights into the level of neuromuscular development (Deschenes et al., 2002; Gillen et al., 2019). The muscles of the upper limbs and shoulder girdle are involved in all types of tennis strokes, and the repeated execution of these movements during long-term training leads to neuromuscular adaptations characteristic of highly skilled tennis players (Julienne et al., 2012).

One of the muscles showing the most pronounced differences in neuromuscular coordination related to tennis training was the deltoid muscle, particularly its posterior part (the posterior deltoid). The posterior deltoid is engaged during all types of tennis strokes, playing varying roles depending on the stroke. For example, during the serve, it is activated in the later phase of the service motion, specifically the cocking phase, just before the acceleration phase (Kibler et al., 2007). In the forehand stroke, the posterior deltoid contracts concentrically during the preparation phase and eccentrically during the follow-through (Reid et al., 2003). In contrast, this pattern is reversed in the backhand stroke (Koronas and Koutlianos, 2021). Moreover, during the backhand volley, the posterior deltoid is activated to a greater extent, up to approximately 50% of MVIC, compared to the forehand volley (Chow et al., 2007).

In the present study, the activity of the posterior deltoid muscle was higher in young female tennis players compared to untrained peers during both ER, where it acts as an agonist, and IR, where it functions as an antagonist, regardless of age group. This difference was particularly evident during contractions at 50% of MVIC, a level known to engage a greater number of motor units than 20% of MVIC. It has been proposed that speed- and strength-oriented athletic training during childhood and adolescence can potentially facilitate the neuromuscular development of fast-fatigue type II motor units (Dotan et al., 2012; Woods et al., 2023). Studies on adult tennis players suggest that the posterior deltoid is especially susceptible to early onset of fatigue (Gaudet et al., 2018). These findings may reflect more advanced neuromuscular coordination, which could partly explain the higher RTD values observed during ER in young tennis players compared with untrained controls.

Current evidence remains inconclusive as to whether agonist NRMS during submaximal contractions (e.g., at 20% or 50% MVIC) differs significantly between trained and untrained individuals (Duchateau et al., 2006; Del Vecchio et al., 2019; Tourino et al., 2020; Skarabot et al., 2021). However, studies on resistance training have shown that antagonist coactivation tends to be lower in both youth (Kochanowicz et al., 2019) and adult athletes (Tillin et al., 2011; Balshaw et al., 2019) with strength- and speed-oriented profiles. However, this effect may be specific to the muscle or joint involved (Bazzucchi et al., 2008; Kochanowicz et al., 2018).

Forehand strokes are typically used more frequently than backhand strokes in competitive tennis (Fernandez-Fernandez et al., 2010; Reid et al., 2016). Together with the increased IR/ER strength ratio, this may suggest that the elevated posterior deltoid activity represent an adaptive response supporting glenohumeral joint stability. Similarly, the increased CI of the infraspinatus muscle during IR in tennis players may reflect the same stabilizing mechanism—particularly during the follow-through phase of the forehand stroke (Ryu et al., 1988; Escamilla and Andrews, 2009). On the other hand, tennis players demonstrated a lower CI of the pectoralis major muscle, which may be associated with the higher RTD values observed during ER. Previous studies have suggested that reduced coactivation of antagonist muscles can contribute to enhanced RTD performance (Valkeinen et al., 2002; Pereira and Goncalves, 2011).

Moreover, in the present study, the CI of the anterior deltoid muscle during ER was higher in untrained girls aged 13–14 compared to those aged 11–12. Conversely, a trend toward lower CI values was observed in the older group of tennis players, which may indicate training-related neuromuscular adaptations contributing to enhanced RTD during ER.

Although the available evidence remains inconclusive, it suggests that with age, the muscle activity of both agonists (Dotan et al., 2012) and antagonists (Woods et al., 2023) during submaximal tasks tends to decrease. This phenomenon may vary depending on the specific muscle or joint involved. In the current study, in addition to the observed increase in anterior deltoid antagonist activity among untrained girls aged 13–14 years, we also found higher activation of the latissimus dorsi muscle in this age group, regardless of training status. Although no significant differences in IR muscle strength normalized to body mass were found between age groups, these differences in muscle activation may reflect an increased IR/ER strength ratio in older participants.

### Limitations

The main limitation of this study lies in its cross-sectional design, which makes it difficult to definitively attribute the observed differences solely to tennis training. It is possible that some of the effects are related to the early selection of children with specific athletic predispositions rather than training itself. However, the study clearly demonstrated that female tennis players with comparable training backgrounds but differing in age and training experience showed distinct performance outcomes, both among themselves and in comparison to untrained peers with similar anthropometric characteristics.

An additional methodological limitation is that neuromuscular performance was assessed under isometric conditions, which do not fully reproduce the dynamic, multi-joint, and high-velocity nature of tennis strokes. Nevertheless, these measures remain functionally relevant because rapid torque generation and shoulder rotational strength contribute to upper-limb control and force transfer during the preparatory and acceleration phases of tennis strokes (Baiget et al., 2016, Baiget et al., 2022).

It should also be acknowledged that the study included only female participants. This was intentional in order to maintain a consistent coaching approach and training methodology across age groups, which would not have been feasible if boys had been included. Each age group was coached by the same coaching staff, ensuring training continuity. Due to this fact, the presented sample size (n = 67) is relatively small considering the four analyzed groups. Thus, while the study meets the criteria for the required total sample size to detect only large effects (n = 52), the actual power was high (0.9).

A key limitation of this study is that biological maturation was not directly assessed. The pubertal period, characterized by significant hormonal changes, may have influenced the development of muscle strength and neuromuscular coordination in girls; therefore, its potential contribution to the observed differences cannot be fully excluded. Nonetheless, as previously noted, the untrained groups did not differ significantly from the trained athletes in terms of body mass or height. Since somatic development associated with puberty is directly linked to increases in muscle strength (Costa et al., 2021), the confounding effect of pubertal maturation on the measured outcomes is likely reduced, although not eliminated.

## Conclusions

Female tennis players aged 11–14 years exhibit a specific glenohumeral neuromuscular profile compared with untrained peers, with the most consistent differences related to higher maximal strength and higher rapid torque production during ER and selected patterns of shoulder muscle activation and coactivation. The age-specific difference observed in 11–12-year-old players suggests that tennis participation may be associated with more advanced development of rapid ER function at an early stage of the training pathway, although the cross-sectional design precludes causal inference.

The SEMG findings further indicate that these differences are not limited to torque-generating capacity, but also involve neuromuscular coordination strategies relevant to glenohumeral control. In particular, altered posterior deltoid activation, pectoralis major and infraspinatus coactivation may reflect function-specific adaptations supporting external-rotator performance and shoulder stabilization during tennis-specific demands.

Overall, these findings suggest that long-term tennis training may be associated with specific neuromuscular characteristics of the glenohumeral joint in young female players. From a practical perspective, these observations may inform age-specific strength and conditioning strategies aimed at monitoring shoulder rotator development, individualizing training loads, and identifying functional imbalances in IR/ER strength during long-term tennis training.

## Data Availability

The raw data supporting the conclusions of this article will be made available by the authors, without undue reservation.
